# Association of Oncologist Participation in Medicare’s Oncology Care Model With Patient Receipt of Novel Cancer Therapies

**DOI:** 10.1001/jamanetworkopen.2022.34161

**Published:** 2022-09-29

**Authors:** Christopher R. Manz, Angela C. Tramontano, Hajime Uno, Ravi B. Parikh, Justin E. Bekelman, Deborah Schrag

**Affiliations:** 1 Division of Population Sciences, Department of Medical Oncology, Dana-Farber Cancer Institute, Boston, Massachusetts; 2 Department of Medical Oncology, Harvard Medical School, Boston, Massachusetts; 3 Department of Medical Ethics and Health Policy, Perelman School of Medicine, University of Pennsylvania, Philadelphia; 4 Penn Center for Cancer Care Innovation, Abramson Cancer Center, University of Pennsylvania, Philadelphia; 5 Corporal Michael J. Crescenz Department of Veterans Affairs Medical Center, Philadelphia, Pennsylvania; 6 Department of Medicine, Memorial Sloan Kettering Cancer Center, New York, New York

## Abstract

**Importance:**

Medicare’s Oncology Care Model (OCM) was an alternative payment model that tied performance-based payments to cost and quality goals for participating oncology practices. A major concern about the OCM regarded inclusion of high-cost cancer therapies, which could potentially disincentivize oncologists from prescribing novel therapies.

**Objective:**

To examine whether oncologist participation in the OCM changed the likelihood that patients received novel therapies vs alternative treatments.

**Design, Setting, and Participants:**

This cohort study of Surveillance, Epidemiology, and End Results (SEER) Program data and Medicare claims compared patient receipt of novel therapies for patients treated by oncologists participating vs not participating in the OCM in the period before (January 2015-June 2016) and after (July 2016-December 2018) OCM initiation. Participants included Medicare fee-for-service beneficiaries in SEER registries who were eligible to receive 1 of 10 novel cancer therapies that received US Food and Drug Administration approval in the 18 months before implementation of the OCM. The study excluded the Hawaii registry because complete data were not available at the time of the data request. Patients in the OCM vs non-OCM groups were matched on novel therapy cohort, outcome time period, and oncologist specialist status. Analysis was conducted between July 2021 and April 2022.

**Exposures:**

Oncologist participation in the OCM.

**Main Outcomes and Measures:**

Preplanned analyses evaluated patient receipt of 1 of 10 novel therapies vs alternative therapies specific to the patient’s cancer for the overall study sample and for racial subgroups.

**Results:**

The study included 2839 matched patients (760 in the OCM group and 2079 in the non-OCM group; median [IQR] age, 72.7 [68.3-77.6] years; 1591 women [56.0%]). Among patients in the non-OCM group, 33.2% received novel therapies before and 40.1% received novel therapies after the start of the OCM vs 39.9% and 50.3% of patients in the OCM group (adjusted difference-in-differences, 3.5 percentage points; 95% CI, −3.7 to 10.7 percentage points; *P* = .34). In subgroup analyses, second-line immunotherapy use in lung cancer was greater among patients in the OCM group vs non-OCM group (adjusted difference-in-differences, 17.4 percentage points; 95% CI, 4.8-30.0 percentage points; *P* = .007), but no differences were seen in other subgroups. Over the entire study period, patients with oncologists participating in the OCM were more likely to receive novel therapies than those with oncologists who were not participating (odds ratio, 1.47; 95% CI, 1.09-1.97; *P* = .01).

**Conclusions and Relevance:**

This study found that participation in the OCM was not associated with oncologists’ prescribing novel therapies to Medicare beneficiaries with cancer. These findings suggest that OCM financial incentives did not decrease patient access to novel therapies.

## Key Points

**Question:**

Was implementation of Medicare’s Oncology Care Model in 2016 associated with a decrease in patient receipt of novel cancer therapies?

**Findings:**

This cohort study included 2839 patients with cancer who were eligible to receive a novel cancer therapy. The start of the Oncology Care Model did not correspond with a decreased likelihood of receiving a novel therapy.

**Meaning:**

Results of this study suggest that participation in the Oncology Care Model was not associated with decreased prescribing of novel cancer therapies for Medicare beneficiaries with cancer.

## Teaser

This cohort study assesses the association between oncologists’ participation in Medicare’s Oncology Care Model and their use of novel cancer therapies for patients with cancer.

## Introduction

Cancer is the second leading cause of death in the United States and accounted for more than $200 billion in annual spending in 2020.^1,2^ In an effort to improve care quality and reduce costs, the Centers for Medicare & Medicaid Services implemented a voluntary alternative payment plan called the Oncology Care Model (OCM) in 2016.^3^ More than 3200 oncology clinicians in 200 practices volunteered to participate in the OCM. The program reimbursed practices with fixed per-patient monthly payments to enhance cancer coordination and delivery services and to improve care quality. Practices were eligible to receive incentive payments for meeting quality metrics and reducing costs below risk-adjusted thresholds for 6-month episodes of care.^3,4^

One concern that arose in the development of the OCM was how to account for the cost of expensive, novel anticancer drugs in the cost thresholds used to ascertain incentive payments.^3^ Target cost thresholds in the OCM were based on the costs that were borne by Medicare (all Part A and B services and, for Part D beneficiaries, a portion of low-income subsidies and catastrophic costs) during the baseline period of 2012 to 2014, which were risk adjusted and projected forward and then discounted to impose cost-saving restraints. However, novel cancer therapies are increasingly expensive, and clinicians have little control of the costs of drugs, which compose more than 50% of cancer care costs, raising concerns that the cost thresholds may discourage use of novel therapies, especially given evidence that financial incentives can affect oncologist prescribing patterns.^5,6^ Thus, Medicare also included a novel therapy adjustment that offset up to 80% of the incremental spending on the use of novel therapies compared with baseline costs in its cost threshold calculations.^3^

Although modeling suggested that this adjustment was sufficient to account for the costs of novel therapies,^7^ early experiences raised concerns that novel therapies were affecting costs that exceeded OCM spending targets.^8,9^ A study found that the OCM was associated with reduced use of expensive supportive care medications.^10^ Recently, a large difference-in-differences analysis of cancer care cost and use under the OCM found that the OCM was not associated with the use of novel chemotherapies for 5 cancers in the first 3 years of the OCM, but the study found an increase in the use of immunotherapies for lung cancer and melanoma (2.5 and 2.9 percentage point difference, respectively) but not for kidney cancer.^6^ However, the study calculated novel therapy use rates for the entire study population by each cancer type rather than subgroups eligible to receive novel therapies, possibly missing important differences in novel therapy use attributable to the OCM. By linking OCM participation data with Surveillance, Epidemiology, and End Results (SEER) Program data, it is possible to better identify patients who would have been candidates for specific types of novel therapy.

To extend previous work and provide a granular analysis of the association between the OCM and patients’ receipt of novel therapies, this study used SEER registry data linked to Medicare enrollment and claims data to identify patients likely to be eligible for novel therapies based on cancer histologic subtype, stage, and treatment history. The aim of the study was to assess the association between oncologists’ participation in the OCM and patients’ receipt of novel therapies using a difference-in-differences approach.

## Methods

### Study Design and Data

This retrospective, registry-based cohort study used SEER registry data linked to Medicare enrollment and fee-for-service claims (SEER-Medicare) from 2014 to 2018 to identify patients potentially eligible to receive 1 of 10 novel cancer therapies and ascertained whether those patients received the novel therapy or an alternative treatment. The study used a nonrandomized difference-in-differences design to evaluate the association between oncologists’ participation in the OCM and patients’ likelihood of receiving a novel cancer treatment in the period before and after the OCM started. The study excluded the Hawaii registry because complete data were not available at the time of the data request. The Dana-Farber Cancer Institute Institutional Review Board deemed the study exempt from review and waived the requirement for written informed consent because it did not involve human research. We followed the Strengthening the Reporting of Observational Studies in Epidemiology (STROBE) reporting guideline.

### Identification of Novel Therapies

To identify novel therapies for inclusion in this study that would have been available to patients both before and after the start of the OCM, authors reviewed a list of all cancer therapies (administered orally or intravenously) that received US Food and Drug Administration approval for a new indication in the 18 months before the OCM’s start on July 1, 2016 (drugs approved before this period were not designated as novel by the OCM). Therapies were included if (1) a cohort of patients eligible for the novel therapy could be identified using SEER-Medicare claims data and (2) the novel therapy had an existing alternative treatment (as described in contemporaneous National Comprehensive Cancer Network guidelines) such that there was a choice to be made between the novel vs alternative therapy, which was observable in claims data. Table 1 describes the 10 new drug indications for patient cohorts included in the study, and eTable 1 in the Supplement has additional details on inclusion and exclusion criteria and how sequentially approved novel therapies were incorporated into the cohorts.

**Table 1.** Novel Therapy Cohorts

Cohort	Novel therapy, date of approval^**a**^	New indication approval^**b**^	Alternative therapies
Breast cancer; ER/PR positive, *ERBB2* negative, first-line therapy	Palbociclib, 2/3/15	First-line therapy with letrozole	Anastrozole, exemestane, everolimus, fulvestrant, letrozole, tamoxifen
Breast cancer; ER/PR positive, *ERBB2* negative, second-line therapy	Palbociclib, 2/19/16 Abemaciclib, 9/28/17	Second-line therapy after disease progression on endocrine therapy	If not part of first-line therapy, anastrozole, exemestane, everolimus, fulvestrant, letrozole, tamoxifen
Colon cancer	Ramucirumab, 4/24/15	Second-line therapy with FOLFIRI, after first-line therapy with FOLFOX-bevacizumab	FOLFIRI given without ramucirumab
Melanoma	Nivolumab, 12/22/14 Pembrolizumab, 12/18/15 Ipilimumab plus nivolumab, 10/30/15	First-line therapy	Ipilimumab monotherapy
Non–small cell lung cancer			
Anaplastic lymphoma kinase positive	Alectinib, 12/11/15	Second-line therapy after progression or intolerance to crizotinib	Docetaxel, etoposide, gemcitabine, paclitaxel, pemetrexed, vinorelbine
Epidermal growth factor receptor positive	Osimertinib, 11/13/15	Second-line therapy with *T790M* sequence variation.	Erlotinib or afatinib (if not first-line therapy); docetaxel, etoposide, gemcitabine, nab-paclitaxel, paclitaxel, pembrolizumab, pemetrexed, vinorelbine
Programmed cell death 1 ligand 1 >50%	Nivolumab, 3/14/15 (squamous histology only), 10/9/15 (all histologies) Pembrolizumab, 10/2/15	Second-line therapy after platinum chemotherapy	If not part of first-line therapy, docetaxel, etoposide, gemcitabine, paclitaxel, pemetrexed, vinorelbine
Pancreatic cancer	Liposomal irinotecan, 10/22/15	Second-line therapy after gemcitabine-containing regimen	Irinotecan, oxaliplatin
Renal cell carcinoma	Lenvatinib, 5/13/16 Cabozantinib, 4/25/16 Nivolumab, 11/23/15	Second-line therapy after antiangiogenesis therapy	If not part of first-line therapy, aldesleukin (IL-2), axitinib, bevacizumab, everolimus, pazopanib, sorafenib, sunitinib, temsirolimus
Urothelial cancer	Atezolizumab, 5/18/16 Nivolumab, 2/2/17 Durvalumab, 5/1/17 Avelumab, 5/9/17 Pembrolizumab, 5/18/17	Metastatic cancer: second-line therapy after platinum chemotherapy. Adjuvant/neoadjuvant treatment: second-line therapy after platinum chemotherapy with disease progression within 12 mo	If not part of first-line therapy, docetaxel, doxorubicin, gemcitabine, ifosfamide paclitaxel

Abbreviations: ER, estrogen receptor; *ERBB2*, erb-B2 receptor tyrosine kinase 2; FOLFIRI, fluorouracil, leucovorin, and irinotecan; FOLFOX, fluorouracil, leucovorin, and oxaliplatin; PR, progesterone receptor.

^a^ Cohort start date is the earliest novel therapy approval date. Subsequent novel therapies may be included starting on the date that the subsequent therapy receives US Food and Drug Administration approval for the same indication. Cohort end dates are 2 years from approval of the earliest novel therapy.

^b^ All indications are for the treatment of metastatic cancer unless otherwise specified. See eTable 1 in the Supplement for additional details.

### Study Populations and Outcomes

All patients who met inclusion criteria for 1 of the novel drugs were assigned to the respective novel drug cohort (eg, non–small cell lung cancer: anaplastic lymphoma kinase positive; eTable 1 in the Supplement). The study outcome was the first claim for the novel therapy vs alternative therapy as long as the claim date was within 2 years of the novel therapy’s Food and Drug Administration approval for use in the applicable cohort. For instance, patients in the anaplastic lymphoma kinase–positive non–small cell lung cancer cohort, which was assessed for the receipt of second-line alectinib therapy, had to have a diagnosis of stage IIIB, IIIC, or IV non–small cell lung cancer; received first-line crizotinib; and received a second-line therapy within 2 years of alectinib’s Food and Drug Administration approval of December 11, 2015. Outcomes were categorized as receipt of novel therapy if the patient received second-line therapy with alectinib or categorized as receipt of alternative therapy if patients received 1 of the chemotherapies listed in eTable 1 in the Supplement. In addition, the outcome date was assigned to the first claim for the outcome (ie, the date of the first claim for either the novel therapy or alternative therapy). Patients also had to have continuous Medicare Parts A, B, and D coverage for 3 months before their diagnosis until the date of their outcome to be included in the analysis. Patients enrolled in Medicare Advantage plans were excluded because their treatment claims were not reliably available.

### Identification of OCM Participation

Oncologists participating in the OCM can bill for a Monthly Enhanced Oncology Services payment for each patient treated under the OCM. A list of OCM participants was generated by finding all National Provider Identifiers (NPIs) associated with these payment claims (Healthcare Common Procedure Coding System code G9678) for patients with a diagnosis of any of the cancers included in this study between the start of the OCM in July 2016 and December 2018. Oncology Care Model participation was categorized at the patient level based on whether the prescriber NPI for the first claim of the outcome treatment was in this encrypted list of oncologists participating in the OCM. In addition, a sensitivity analysis was performed using a list of NPIs (converted to encrypted NPIs by SEER-Medicare data managers [R.B.P. and J.E.B]) for oncologists participating in the OCM who were identified in a previous publication by searching the websites of OCM-participating practices in late 2017.^11^

### Statistical Analysis

Data were analyzed between July 2021 and April 2022. χ^2^ and Kruskal-Wallis tests were used to compare unmatched patients in the OCM and non-OCM groups based on measured characteristics, including age, sex, race, ethnicity, marital status, Charlson Comorbidity Index score, disease cohort, urbanicity, Census tract poverty rate, and treating oncologist. To adjust for observable baseline differences between the OCM and non-OCM groups, greedy matching up to a 3:1 ratio was used to exactly match on novel therapy cohort (ie, patients must be assigned to the same novel therapy cohort described in Table 1), outcome date in 6-month increments (eg, first half of 2016), and clinician specialty status. Previous research has found differences in characteristics of clinicians participating vs not participating in the OCM, but such characteristics were not available for matching because of the encrypted NPIs.^11^ Instead, specialist status was inferred by examining all of the claims for each NPI in the study data, calculating the percentage of the total number of unique patients for each NPI that had an *International Statistical Classification of Diseases and Related Health Problems, Tenth Revision* code for each disease category (bladder, breast, gastrointestinal [colon and pancreas], melanoma, lung, and renal). Clinicians for which 50% or more of patients had 1 disease category were categorized as specialists, and those without any categories of 50% or more were categorized as generalists; clinicians with fewer than 5 patients were categorized as low volume.

In addition to a descriptive summary of findings, a multivariable mixed-effects regression model with random effects for matching group and logit link was used to calculate patient likelihood of receipt of a novel therapy compared with alternative therapies in the 2 years after Food and Drug Administration approval, with OCM participation as the projected variable and the interaction of the OCM and receipt of treatment after the start of the OCM on July 1, 2016, as the outcome of interest. Because matching was not expected to balance characteristics across all measured variables, the models also included the following covariates: categorical age, sex, race, Hispanic ethnicity, marital status, Census tract poverty rate, and urbanicity (metropolitan vs nonmetropolitan area), which were all reported in the SEER registry, plus Charlson Comorbidity Index score (0 indicates no comorbid conditions; a higher score indicates an increasing number or severity of comorbid conditions), novel therapy cohort, time period, and specialist status.^12^ Race and ethnicity were included to evaluate secondary hypotheses regarding disparities in access to novel therapies and were defined as coded by the SEER registry. Using the estimated parameters of the mixed-effects model, the between-group difference (ie, difference-in-differences) and corresponding 95% CI were derived. Inclusion of registry and state were not permitted by the data use agreement to prevent inadvertent identification of OCM practices. The parallel baseline trends assumption was tested using the same model restricted to observations before the start of the OCM and including 3-month time periods and an interaction between these time periods and the OCM to test the trend. Patients with missing values for any of the variables included in the model were excluded from the analysis with the mixed-effects model (n <11 patients [<1% of study sample]). A prespecified analysis to assess differences between Black and White patients in the between-group difference (ie, difference-in-difference-in-differences) used the same model as the primary analysis and added the following 3 interaction terms: race by OCM group, race by post-OCM start, and race by OCM group and post-OCM start.

A 2-sided α < .05 was the threshold for statistical significance; a confidence β coefficient of 0.95 was used to calculate 95% CIs. Analysis was performed using SAS Enterprise Guide, version 7.15 (SAS Institute Inc) and Stata/IC, version 15.1 (StataCorp LLC).

## Results

The unmatched study sample included 3310 patients, which decreased to 2839 patients (median [IQR] age, 72.7 [68.3-77.6] years; 1591 women [56.0%] and 1248 men [44.0%]) after matching, with 760 patients in the OCM group and 2079 in the non-OCM group. Matching increased the similarities between the groups for the matched variables of the cohort (unmatched vs matched standardized difference, 0.16 vs 0.05), time period (unmatched vs matched standardized difference, 0.10 vs 0.04), and treating oncologist (unmatched vs matched standardized difference, 0.14 vs 0.04) (eTables 2 and 3 in the Supplement). Among matched patients, 181 (6.4%) were Asian or Pacific Islander individuals, individuals of other race (including Alaska Native or American Indian or indicated as other in the SEER registry), or of unknown race; 232 were Black individuals (8.2%); and 2426 were White individuals (85.5%). One hundred eighty-four individuals (6.5%) had Hispanic ethnicity and 2655 (93.5%) had non-Hispanic ethnicity. A total of 1740 patients (61.3%) were treated by oncology generalists (Table 2). Matched patients eligible to receive second-line immunotherapy for metastatic non–small cell lung cancer (n = 994 [35.0%]), second-line liposomal irinotecan for pancreatic cancer (n = 753 [26.5%]), and first-line palbociclib for metastatic hormone receptor–positive erb-B2 receptor tyrosine kinase 2 (*ERBB2*; formerly *HER2*)–negative breast cancer (n = 376 [13.2%]) composed the 3 largest novel therapy cohorts. Unmatched patients treated by clinicians participating in the OCM were more often White individuals (672 of 764 [88.0%] in the OCM group vs 2143 of 2546 [84.2%] in the non-OCM group; *P* = .04), lived in a metropolitan area (687 of 764 [89.9%] in the OCM group vs 2130 of 2546 [83.7%] in the non-OCM group; *P* < .001), and lived in a low Census poverty track (0% to <5% poverty rate, 232 of 764 [30.4%] in the OCM group vs 577 of 2546 [22.7%] in the non-OCM group; *P* < .001) (eTable 2 in the Supplement). A total of 1819 clinicians had a median of 1 patient in the matched sample (IQR, 25%-75% of 1:1 matched sample).

**Table 2.** Demographic Characteristics

Characteristic	No. (%)				
	Total (N = 2839)	Non-OCM group		OCM group	
		Before intervention (n = 930)	After intervention (n = 1149)	Before intervention (n = 348)	After intervention (n = 412)
Age at outcome, median (IQR), y	72.7 (68.3-77.6)	72.3 (68.1-77.6)	72.5 (68.3-77.7)	73.4 (68.9-77.3)	72.8 (68.5-77.4)
Sex					
Female	1591 (56.0)	543 (58.4)	612 (53.3)	210 (60.3)	226 (54.9)
Male	1248 (44.0)	387 (41.6)	537 (46.7)	138 (39.7)	186 (45.1)
Race					
Asian, Pacific Islander, Other, and unknown^a^	181 (6.4)	62 (6.7)	86 (7.5)	16 (4.6)	17 (4.1)
Black	232 (8.2)	91 (9.8)	84 (7.3)	29 (8.3)	28 (6.8)
White	2426 (85.5)	777 (83.5)	979 (85.2)	303 (87.1)	367 (89.1)
Ethnicity					
Hispanic	184 (6.5)	55 (5.9)	79 (6.9)	21 (6.0)	29 (7.0)
Non-Hispanic	2655 (93.5)	875 (94.1)	1070 (93.1)	327 (94.0)	383 (93.0)
Marital status					
Unmarried	970 (34.2)	335 (36.0)	386 (33.6)	106 (30.5)	143 (34.7)
Married	1504 (53.0)	499 (53.7)	608 (52.9)	197 (56.6)	200 (48.5)
Unknown	365 (12.9)	96 (10.3)	155 (13.5)	45 (12.9)	69 (16.7)
Charlson Comorbidity Index score^b^^,^^c^					
0	1091 (38.4)	380 (40.9)	416 (36.2)	144 (41.4)	151 (36.7)
1	820-830 (28.9-29.2)	270-280 (29.0-30.1)	320-330 (27.9-28.7)	90-100 (25.9-28.7)	127 (30.8)
>2	918 (32.3)	272 (29.2)	409 (35.6)	103 (29.6)	134 (32.5)
Missing	<11 (0-0.4)	<11 (0-1.0)	<11 (0-0.9)	<11 (0-2.9)	0
Cohort^c^					
Lung					
Anaplastic lymphoma kinase	12 (0.4)	<11 (0-1.1)	<11 (0-0.9)	<11 (0-2.9)	<11 (0-2.4)
Epidermal growth factor receptor	113 (4.0)	33 (3.5)	51 (4.4)	11 (3.2)	18 (4.4)
Second-line immunotherapy	994 (35.0)	384 (41.3)	348 (30.3)	140 (40.2)	122 (29.6)
Bladder	105 (3.7)	0	77 (6.7)	0	28 (6.8)
Pancreas	753 (26.5)	183 (19.7)	368 (32.0)	76 (21.8)	126 (30.6)
Colon	181 (6.4)	93 (10.0)	41 (3.6)	32 (9.2)	15 (3.6)
Kidney	155 (5.5)	10-20 (1.1-2.2)	103 (9.0)	<11 (0-2.9)	36 (8.7)
Breast					
First line	376 (13.2)	187 (20.1)	86 (7.5)	68 (19.5)	35 (8.5)
Second line	91 (3.2)	10-20 (1.1-2.2)	54 (4.7)	<11 (0-2.9)	22 (5.3)
Melanoma	59 (2.1)	21 (2.3)	10-20 (0.9-1.7)	11 (3.2)	<11 (0-2.4)
Urbanicity					
Metropolitan area	2423 (85.3)	774 (83.2)	965 (84.0)	306 (87.9)	378 (91.7)
Nonmetropolitan area	416 (14.7)	156 (16.8)	184 (16.0)	42 (12.1)	34 (8.3)
Census tract poverty rate, %^d^					
0 to <5	689 (24.3)	188 (20.2)	269 (23.4)	91 (26.1)	141 (34.2)
5 to <10	672 (23.7)	212 (22.8)	266 (23.2)	92 (26.4)	102 (24.8)
10 to <15	801 (28.2)	275 (29.6)	345 (30.0)	90 (25.9)	91 (22.1)
15 to <20	473 (16.7)	192 (20.6)	168 (14.6)	55 (15.8)	58 (14.1)
Unknown	204 (7.2)	63 (6.8)	101 (8.8)	20 (5.7)	20 (4.9)
Treating oncologist^c^					
Low volume	32 (1.1)	12 (1.3)	11 (1.0)	<11 (0-2.9)	<11 (0-2.4)
Specialist	1067 (37.6)	333 (35.8)	437 (38.0)	130-140 (37.4-40.2)	160-170 (38.8-41.3)
Generalist	1740 (61.3)	585 (62.9)	701 (61.0)	211 (60.6)	243 (59.0)

Abbreviation: OCM, Oncology Care Model.

^a^ Other includes Alaska Native or American Indian or indicated as other in the SEER registry.

^b^ Zero indicates no comorbid conditions; a greater score indicates an increasing number or severity of comorbid conditions.

^c^ Ranges are provided for some values to comply with the Centers for Medicare & Medicaid Services cell suppression policy.

^d^ This category indicates the percentage of individuals living in the Census tract who have an income below the federal poverty level.

Patient receipt of novel therapy steadily increased over time (Figure). Baseline trends according to OCM status differed significantly in the unmatched sample (odds ratio for before OCM time trend, 0.73; 95% CI, 0.57-0.93; *P* = .01) but not in the matched sample (odds ratio for time trend, 0.81; 95% CI, 0.62-1.06; *P* = .12). In the multivariable mixed-effects regression analysis, calculated patient likelihood of receipt of novel therapy for those treated by oncologists not participating in the OCM increased from 33.2% before July 2016 to 40.1% after July 2016 compared with an increase from 39.9% to 50.3% over the same period for patients treated by participating oncologists, a nonsignificant difference-in-differences of 3.5 percentage points (95% CI, −3.7 to 10.7 percentage points; *P* = .34). When the novel drug cohorts were analyzed independently, OCM participation was associated with an increase in patient receipt of novel therapy only for second-line immunotherapy for lung cancer (17.4 percentage points; 95% CI, 4.8-30.0 percentage points; *P* = .007). The other cohorts showed no significant differences in receipt of novel therapy or had sample sizes that were too small to run the model.

**Figure.**
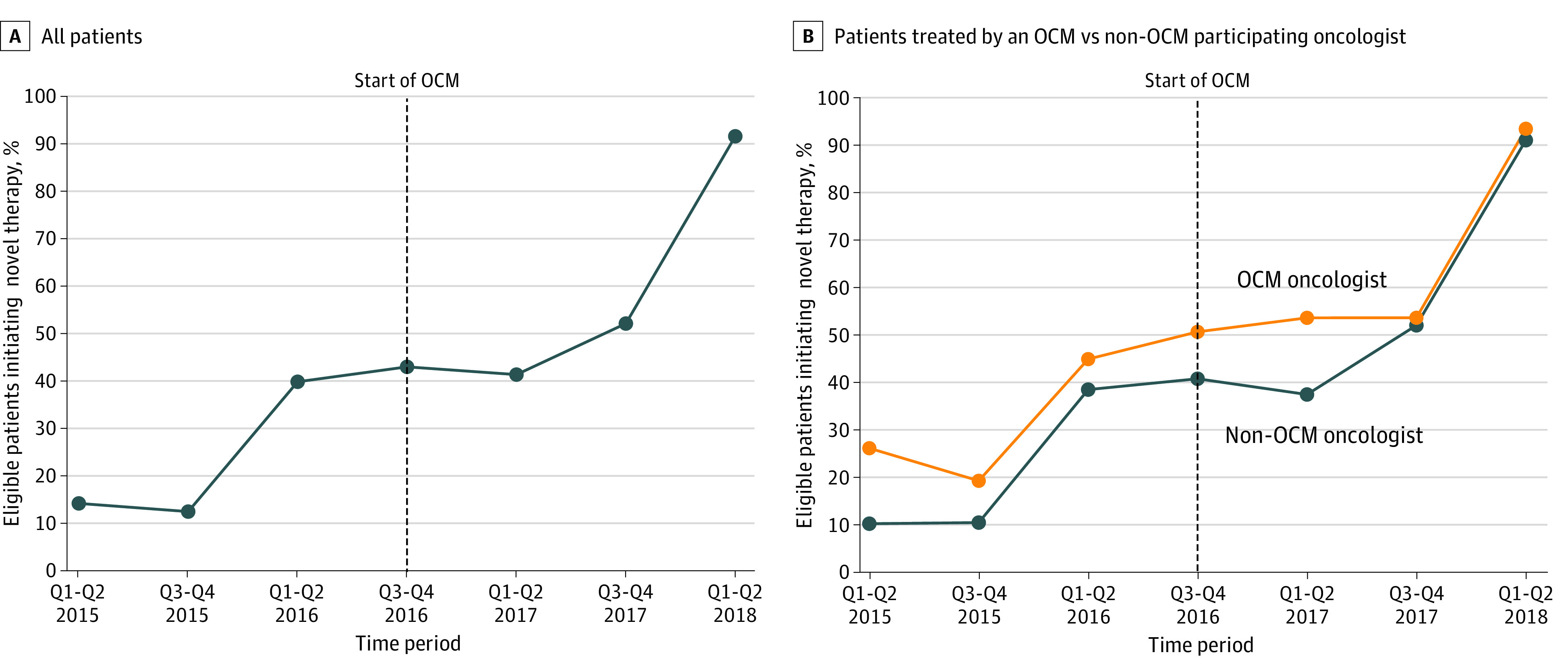
Percent of Eligible Patients Receiving a Novel Therapy From January 2015 Through June 2018 OCM indicates Oncology Care Model; Q, quarter.

Among all cohorts, patients treated by oncologists participating in the OCM were 47% more likely to receive a novel therapy over the entire time period compared with those treated by nonparticipating oncologists (odds ratio, 1.47; 95% CI, 1.09-1.97; *P* = .01). Black patients were 39% less likely to receive novel therapies compared with White patients (odds ratio, 0.61; 95% CI, 0.42-0.89; *P* = .009). Likelihood of receipt of novel therapies among Black patients of clinicians participating in the OCM increased from 27.8% before OCM initiation to 54.1% after its implementation vs an increase of 40.8% before OCM initiation to 49.9% after implementation for comparable White patients. In addition, this group was the only group of Black patients to have higher rates of novel therapy receipt than comparable White patients (eTable 4 in the Supplement). In contrast, disparity in receipt of novel therapies increased for Black patients (vs White patients) of nonparticipating OCM practices after OCM implementation. The difference-in-differences estimate for the OCM group was 23.0 percentage points (95% CI, −2.4 to 48.5 percentage points; *P* = .08) for Black patients and 1.8 percentage points (95% CI, −6.0 to 9.5 percentage points; *P* = .66) for White patients. A preplanned difference-in-difference-in-differences analysis showed no association for the OCM on the difference in this Black/White racial disparity (difference-in-difference-in-differences, 21.2 percentage points; 95% CI, −5.5 to 48.0 percentage points; *P* = .12). Parallel trend and regression results are available in eAppendixes 1 through 4 in the Supplement. A sensitivity analysis that used the alternative method of identifying oncologists participating in the OCM showed similar results (eAppendix 5 in the Supplement).

## Discussion

This study analyzed patient cohorts based on cancer site, stage, and treatment history using SEER-Medicare data to measure the association between receiving treatment from an oncologist participating in the OCM and receipt of novel cancer therapies. Across a range of cancer types, no observable association between physician participation in the OCM and the likelihood of patient receipt of novel therapies was found. However, patients treated in OCM-participating practices were more likely to receive novel therapies both before and after initiation of the OCM. After OCM implementation, the likelihood of receiving novel therapy for second-line treatment of advanced lung cancer was higher for patients treated at OCM-participating practices.

Some single-practice reports suggested that patients who receive standard of care treatment with novel therapies, including patients with lung cancer treated with immunotherapy, had health care spending that exceeded OCM-defined cost thresholds and could theoretically alter physician prescribing of novel therapies.^8,9^ However, another study that used broader cohort definitions and ascertained eligibility for novel therapies based solely on diagnosis with a cancer for which a novel therapy is approved found either no association (for chemotherapy for 5 cancers and immunotherapy for 1 cancer) or an association with limited cancer types (for immunotherapy for 2 cancers).^6^ The current study used more rigid cohort definitions in identifying patients potentially eligible to receive novel therapies, but because of the smaller sample size, all chemotherapy and immunotherapy novel treatments were combined in 1 analysis, and no statistically significant differences were found in the receipt of novel therapies before and after implementation of the OCM. Despite the concerns raised specifically regarding patients with lung cancer treated with immunotherapy,^8^ this study found an increase in receipt of novel immunotherapy after the start of the OCM for patients treated by participating clinicians, echoing findings from Keating et al.^6^ In combination, the results of these 2 studies may reassure patients, clinicians, and policy makers that the OCM does not appear to discourage the use of novel therapies despite incentives to reduce cancer spending.

Two factors may explain why a financial disincentive to prescribe novel therapies may not translate into a change in prescribing practices. First, the financial disincentive may be successfully attenuated by the novel therapy adjustment, outweighed by the oncologists’ financial benefit from higher drug reimbursement of expensive novel therapies (for IV therapies) or outweighed by the clinical benefit to the patient. Of note, all OCM practices participated in the single-sided risk model (good performance is rewarded but poor performance is not penalized; no practices participated in 2-sided risk)^6^; loss aversion as part of 2-tailed risk participation may have been a more effective disincentive that was avoided by all practices. The earlier study found a decrease in spending on supportive care medications, such as bone-modifying agents and growth factors, suggesting that oncologists may be responsive to this financial incentive if they believe that cheaper alternatives would not compromise patient care.^6^ Second, OCM performance payments and penalties are calculated retrospectively. The current study evaluated novel therapies early in the OCM period before practices received any feedback on cost performance. Oncology Care Model’s retrospective performance measurement that incorporates a practice’s novel therapy use compared with that of all other practices prevents practices from anticipating their performance and adjusting behavior, particularly early in the period after OCM implementation. However, Keating et al^6^ evaluated an extended time period through 2019 and found no association between OCM participation and novel therapy prescribing. These considerations and the results of this study need to inform the implementation and evaluation of the successor to the OCM, the Enhancing Oncology Model, which is expected to start in July 2023.^13^

Although the difference-in-differences approach showed that the OCM was not associated with a decrease in patient receipt of novel therapies as a whole, in adjusted analyses, patients treated by oncologists participating in the OCM were more likely to receive a novel therapy overall and more likely to receive immunotherapy for second-line treatment of lung cancer after the start of the OCM. To reduce confounding, this study matched patients on 3 patient and clinician characteristics and adjusted for many other observed variables, but encrypted NPIs and data use restrictions prevented matching on other unmeasured physician characteristics that may explain this finding. Previous studies have found that oncologists who voluntarily participated in the OCM had different characteristics than nonparticipating physicians^11^ and may be early adopters of new therapies or have different approaches to evaluating the value and utility of new treatments.

The study also found that Black patients were much less likely to receive a novel therapy, a finding consistent with other studies describing racial disparities in access to novel therapies.^14,15,16^ Particularly for cancers for which novel therapies offer substantial improvement in outcomes (eg, immunotherapy for non–small cell lung cancer^17,18^), further research is necessary to identify the underlying causes for persistent disparities in novel technology access and test remedies for closing these gaps. Furthermore, the difference-in-difference-in-differences analysis to assess the association between the OCM and disparities in access to novel therapies showed a large increase in receipt of novel therapies for Black patients associated with the OCM, but the finding was not significant (*P* = .12) in this underpowered prespecified subanalysis. This finding raises the possibility that the OCM might have helped narrow racial disparities in patient access to novel therapies, which would be a noteworthy advance if it bears out in future research. The association between payment models and disparities in access to care needs to be incorporated into evaluation of the OCM’s successor model as well as assessments to identify why the payment model has such an association.

### Limitations

This study has limitations. The difference-in-differences estimate has a wide CI, suggesting that the study is underpowered. The cohort was small because of exacting specifications, but the sample size of individual cohorts was in line with other studies using SEER-Medicare data (eg, receipt of second-line treatment of non–small cell lung cancer^19^), and the results are consistent with a much larger but less selective study that similarly found no association between the OCM and patient receipt of novel therapies.^6^ This analysis was performed at the patient and physician level; the OCM is a practice-level intervention, and physicians may switch between OCM and non-OCM participating practices during the study period, but small sample sizes limit clinician-specific or practice-level analyses. Difference-in-differences analyses for evaluating receipt of novel therapy are also challenging because the definition of novel therapy changes over time, so preintervention and postintervention groups may differ in cohort composition, as we observed in this study. In addition, cohort and treatment definitions were subject to misattribution bias (eg, programmed cell death 1 ligand 1 status is unknown for patients with lung cancer) that may overestimate or underestimate receipt of novel therapy but would not be expected to bias results in the direction of either OCM or non-OCM participation.

## Conclusions

Despite concerns that the OCM inadequately considers the costs of novel therapies in calculating episode payments, the OCM had no discernable association with the overall likelihood that patients receive novel cancer therapies. In conjunction with previous studies, these results can inform the development and evaluation of cost and use incentives for Medicare’s new Enhancing Oncology Model.

## Notes

Supplement. **eTable 1.** Detailed Cohort Definitions **eTable 2.** Demographic Characteristics by Oncology Care Model Status—Unmatched Sample **eTable 3.** Demographic Characteristics by Oncology Care Model Status—Matched Sample **eTable 4.** Receipt of Novel Therapies by Oncology Care Model Status, Before and After Intervention, Black vs White Patients **eAppendix 1.** Parallel Trends Regression Output **eAppendix 2.** Difference-in-Differences Regression Output **eAppendix 3.** Difference-in-Differences Regression Output for Second-Line Immunotherapy in Lung Cancer Cohort **eAppendix 4.** Difference-in-Difference-in-Differences Regression Output for Race **eAppendix 5.** Regression Output for Sensitivity Analysis of Primary Difference-in-Differences Model Using Alternative Specification of Oncology Care Model Practices
