# Supplementary material for: Association of Oncologist Participation in Medicare’s Oncology Care Model With Patient Receipt of Novel Cancer Therapies
Source: JAMA Netw Open. 2022 Sep 29;5(9):e2234161. doi: 10.1001/jamanetworkopen.2022.34161 (PMC9523492; doi:10.1001/jamanetworkopen.2022.34161)
Supplement: Supplement. — eTable 1. Detailed Cohort Definitions eTable 2. Demographic Characteristics by Oncology Care Model Status—Unmatched Sample eTable 3. Demographic Characteristics by Oncology Care Model Status—Matched Sample eTable 4. Receipt of Novel Therapies by Oncology Care Model Status, Before and After Intervention, Black vs White Patients eAppendix 1. Parallel Trends Regression Output eAppendix 2. Difference-in-Differences Regression Output eAppendix 3. Difference-in-Differences Regression Output for Second-Line Immunotherapy in Lung Cancer Cohort eAppendix 4. Difference-in-Difference-in-Differences Regression Output for Race eAppendix 5. Regression Output for Sensitivity Analysis of Primary Difference-in-Differences Model Using Alternative Specification of Oncology Care Model Practices [file jamanetwopen-e2234161-s001.pdf]

Supplementary Online Content

Manz CR, Tramontano AC, Uno H, Parikh RB, Bekelman JE, Schrag D. Association of oncologist participation in Medicare's Oncology Care Model with patient receipt of novel cancer therapies. *JAMA Netw Open.* 2022;5(9):e2234161.
doi:10.1001/jamanetworkopen.2022.34161

eTable 1. Detailed Cohort Definitions
eTable 2. Demographic Characteristics by Oncology Care Model Status—Unmatched Sample
eTable 3. Demographic Characteristics by Oncology Care Model Status—Matched Sample
eTable 4. Receipt of Novel Therapies by Oncology Care Model Status, Before and After Intervention, Black vs White Patients
eAppendix 1. Parallel Trends Regression Output
eAppendix 2. Difference-in-Differences Regression Output
eAppendix 3. Difference-in-Differences Regression Output for Second-Line Immunotherapy in Lung Cancer Cohort
eAppendix 4. Difference-in-Difference-in-Differences Regression Output for Race
eAppendix 5. Regression Output for Sensitivity Analysis of Primary Difference-in-Differences Model Using Alternative Specification of Oncology Care Model Practices

This supplementary material has been provided by the authors to give readers additional information about their work.

© 2022 Manz CR et al. *JAMA Network Open.*

eTable 1. Detailed cohort definitions

Cohort	Drug names and approval date *	New indication (all metastatic unless indicated)	Cohort definition**	Outcome* Subsequent claim for…
**Breast cancer; ER/PR+, ERBB2 negative, 1st line**	palbociclib 2/3/15	1st line with letrozole	1) Stage IV breast cancer<br>2) SEER: ER or PR positive, HER2 negative	1: palbociclib + letrozole<br>0: anastrozole, exemestane, everolimus, fulvestrant, letrozole without palbociclib within 28 days, tamoxifen
**Breast cancer; ER/PR+, ERBB2 negative, 2nd line**	palbociclib 2/19/16<br>abemacicib 9/28/17	2nd line after disease progression on endocrine therapy	1) Stage IV breast cancer<br>2) SEER: ER or PR positive, HER2 negative<br>3) claim for endocrine therapy – anastrazole, letrozole, exemestane<br>4) no claim for palbociclib, abemaciclib or ribociclib prior to or in conjunction with endocrine therapy	1: palbociclib, abemaciclib (after 9/28/17)<br>0: anastrozole, exemestane, everolimus, fulvestrant alone, letrozole, tamoxifen (*all: if not part of first line therapy*)
**Colon cancer**	ramucirumab 4/24/15	2nd line with FOLFIRI, after 1st line FOLFOX-bevacizumab	1) Stage IV colon cancer<br>2) claims for first line 5-FU, oxaliplatin, bevacizumab<br>3) no preceding claim for irinotecan	1: 5-FU + irinotecan + ramucirumab<br>0: 5-FU + irinotecan
**Melanoma**	nivolumab 12/22/14<br>pembrolizumab 12/18/15<br>ipilimumab + nivolumab 10/30/15	1st line	1) Stage IV melanoma<br>2) No claims for first line treatment with dabrafenib, trametinib, vemurafenib, cobimetinib, binimetinib, encorafenib	1: ipilimumab+nivolumab (starting 10/30/15), pembrolizumab (starting 1/23/16) or nivolumab (starting 12/22/14)<br>0: ipilimumab
**Non-small cell lung cancer: ALK+**	alectinib 12/11/15	2nd line after progression or intolerance to crizotinib	1) Stage IIIB/IIIC/IV NSCLC<br>2) claim for first line crizotinib	1: alectinib<br>0: docetaxel, etoposide, gemcitabine, nab-paclitaxel, paclitaxel, pembrolizumab, pemetrexed, vinorelbine
**Non-small cell lung cancer: EGFR+**	osimertinib 11/13/15	2nd line with T790M mutation	1) Stage IIIB/IIIC/IV NSCLC<br>2) claim for first line erlotinib, gefitinib or afatinib	1: osimertinib<br>0: erlotinib or afatinib (*if not first line therapy*); docetaxel, etoposide, gemcitabine, nab-paclitaxel, paclitaxel, pembrolizumab, pemetrexed, vinorelbine
**Non-small cell lung cancer: PDL1>50%**	nivolumab:<br>3/14/15 (squamous histology only)<br>10/9/15 (all histologies)<br>pembrolizumab 10/2/15	2nd line after platinum chemotherapy	1) Stage IV NSCLC<br>2) claim for first line chemotherapy that includes pemetrexed, paclitaxel, etoposide, docetaxel, gemcitabine, topotecan, nab-paclitaxel, or vinorelbine given with carboplatin or cisplatin	1: nivolumab or pembrolizumab (starting 10/9/15)<br>0: pemetrexed, paclitaxel, etoposide, docetaxel, gemcitabine, topotecan, nab-paclitaxel, vinorelbine (*all: if not part of first line therapy*)
**Pancreatic cancer**	liposomal irinotecan 10/22/15	2nd line after gemcitabine-containing regimen	1) Stage IV pancreatic adenocarcinoma<br>2) claim for first-line gemcitabine	1: liposomal irinotecan +/- 5FU<br>0: irinotecan, oxaliplatin
**Renal cell carcinoma**	lenvatinib 5/13/16<br>cabozantinib 4/25/16<br>nivolumab 11/23/15	2nd line after anti-angiogenesis	1) Primary site cancer of RCC, any stage<br>2) claim for first line axitinib, bevacizumab, pazopanib, sorafenib, sunitinib	1: nivolumab (11/23/15), lenvatinib (after 5/13/16) or cabozantinib (after 4/25/16)<br>0: aldesleukin (IL-2), axitinib, bevacizumab, everolimus, pazopanib, sorafenib, sunitinib, temsirolimus (*all: if not part of first line therapy*)
**Urothelial cancer**	atezolizumab 5/18/16<br>nivolumab 2/2/17<br>durvalumab 5/1/17<br>avelumab 5/9/17<br>pembrolizumab 5/18/17	2nd line after platinum chemotherapy; or 2nd line after platinum chemo for adjuvant/neoadjuvant with disease progression within 12 months	1) Stage = II-IV urothelial cancer<br>2) claim for first line carboplatin or cisplatin	1: atezolizumab, nivolumab (starting 2/2/17) , durvalumab (starting 5/1/17), avelumab (after 5/9/17), pembrolizumab (after 5/18/17)<br>0: docetaxel, doxorubicin, gemcitabine, ifosfamide, methotrexate, paclitaxel (*all: if not part of first line therapy)*<br>*If Stage II-III, outcome claim must be within 12 months of last platinum claim.*

* Some novel therapy cohorts eventually have more than one novel therapy with the same indication. The date of the first novel therapy approval established the two-year evaluation period for the cohort. Patients receiving subsequently-approved novel therapies were included if their first treatment claim occurred after the subsequent novel therapy's FDA approval, as reflected by the dates in the table.

© 2022 Manz CR et al. *JAMA Network Open.*

** All cohort definitions also included: cohort diagnosis is the first reported cancer, continuous Medicare coverage for Parts A/B (and Part D if oral drugs are part of the cohort definition or outcomes) for 3 months prior to diagnosis and until the outcome, and no systemic therapy prior to diagnosis.

© 2022 Manz CR et al. *JAMA Network Open.*

eTable 2. Demographic Characteristics by Oncology Care Model status—unmatched cohort

	Total N=3,310	Non-OCM N=2,546	OCM N=764	Standardized Difference
Median age at outcome, yrs (interquartile range)	72.7 (68.2-77.5)	72.4 (68.2-77.6)	73.1 (68.7-77.3)	-0.08
Sex (% Female)	1,843 (55.7%)	1,406 (55.2%)	437 (57.2%)	0.04
Race				0.14
White	2,815 (85.0%)	2,143 (84.2%)	672 (88.0%)	
Black	268 (8.1%)	210 (8.2%)	58 (7.6%)	
Asian, Pacific Islander, Other and Unknown	227 (6.9%)	193 (7.6%)	34 (4.5%)	
Hispanic	218 (6.6%)	168 (6.6%)	50 (6.5%)	<0.01
Marital status				0.11
Unmarried	1,137 (34.4%)	886 (34.8%)	251 (32.9%)	
Married	1,765 (53.3%)	1,367 (53.7%)	398 (52.1%)	
Unknown	408 (12.3%)	293 (11.5%)	115 (15.1%)	
Charlson comorbidity score				0.06
0	1,281 (38.7%)	985 (38.7%)	296 (38.7%)	
1	930-940 (28.0-29.0%)	700-710 (27.0-28.0%)	220-230 (29.0-30.0%)	
>2	1,084 (32.7%)	847 (33.3%)	237 (31.0%)	
Missing	<11 (0-2%)	<11 (0-2%)	<11 (0-2%)	
Cohort				0.16
Lung- ALK	33 (1.0%)	20-30 (1.0-2.0%)	<11 (0-2%)	
Lung- EGFR	177 (5.3%)	147 (5.8%)	30 (3.9%)	
Lung- 2nd line immunotherapy	1,044 (31.5%)	782 (30.7%)	262 (34.3%)	
Bladder	160 (4.8%)	132 (5.2%)	28 (3.7%)	
Pancreas	817 (24.7%)	615 (24.2%)	202 (26.4%)	
Colon	239 (7.2%)	192 (7.5%)	47 (6.2%)	
Kidney	208 (6.3%)	167 (6.6%)	41 (5.4%)	
Breast- first line	434 (13.1%)	331 (13.0%)	103 (13.5%)	
Breast- second line	109 (3.3%)	83 (3.3%)	26 (3.4%)	
Melanoma	89 (2.7%)	70-80 (2.0-3.0%)	10-20 (2.0-3.0%)	
Time period				0.10
Q1/2 2015	144 (4.4%)	117 (4.6%)	27 (3.5%)	
Q3/4 2015	395 (11.9%)	310 (12.2%)	85 (11.1%)	
Q1/2 2016	964 (29.1%)	726 (28.5%)	238 (31.2%)	
Q3/4 2016	895 (27.0%)	685 (26.9%)	210 (27.5%)	
Q1/2 2017	572 (17.3%)	441 (17.3%)	131 (17.1%)	
Q3/4 2017	286 (8.6%)	228 (9.0%)	58 (7.6%)	
Q1/2 2018	54 (1.6%)	39 (1.5%)	15 (2.0%)	
Urbanicity				0.19
Metro	2,817 (85.1%)	2,130 (83.7%)	687 (89.9%)	
Non-metro	493 (14.9%)	416 (16.3%)	77 (10.1%)	
Census tract poverty rate				0.23
0% - <5%	809 (24.4%)	577 (22.7%)	232 (30.4%)	
5% - <10%	780 (23.6%)	585 (23.0%)	195 (25.5%)	
10% - <155%	908 (27.4%)	725 (28.5%)	183 (24.0%)	
15% - <20%	564 (17.0%)	450 (17.7%)	114 (14.9%)	
Unknown	249 (7.5%)	209 (8.2%)	40 (5.2%)	

© 2022 Manz CR et al. *JAMA Network Open.*

Treating oncologist				0.14
Low volume	91 (2.7%)	80-90 (3.0-2.0%)	<11 (0-2%)	
Specialist	1,215 (36.7%)	910-920 (35.0-36.0%)	290-300 (38.0-39.0%)	
Generalist	2,004 (60.5%)	1,548 (60.8%)	456 (59.7%)	
** Ranges provided for some values to comply with CMS's cell suppression policy.*				

© 2022 Manz CR et al. *JAMA Network Open.*

eTable 3. Demographic Characteristics by Oncology Care Model status—matched cohort

	Total N=2,839	Non-OCM N=2,079	OCM N=760	Standardized Difference
Median age at outcome, yrs (interquartile range)	72.7 (68.3-77.6)	72.4 (68.2-77.7)	73.2 (68.7-77.4)	-0.09
Sex (% Female)	1,591 (56.0%)	1,155 (55.6%)	436 (57.4%)	0.04
Race				0.13
White	2,426 (85.5%)	1,756 (84.5%)	670 (88.2%)	
Black	232 (8.2%)	175 (8.4%)	57 (7.5%)	
Asian, Pacific Islander, Other and Unknown	181 (6.4%)	148 (7.1%)	33 (4.3%)	
Hispanic	184 (6.5%)	134 (6.4%)	50 (6.6%)	<0.01
Marital status				0.09
Unmarried	970 (34.2%)	721 (34.7%)	249 (32.8%)	
Married	1,504 (53.0%)	1,107 (53.2%)	397 (52.2%)	
Unknown	365 (12.9%)	251 (12.1%)	114 (15.0%)	
Charlson comorbidity score				0.03
0	1,091 (38.4%)	796 (38.3%)	295 (38.8%)	
1	820-830 (29.0-30.0%)	590-600 (28.0-29.0%)	220-230 (29.5-30.5%%)	
>2	918 (32.3%)	681 (32.8%)	237 (31.2%)	
Missing	<11 (0-2%)	<11 (0-2%)	<11 (0-2%)	
Cohort				0.05
Lung- ALK	<11 (0-2%)	<11 (0-2%)	<11 (0-2%)	
Lung- EGFR	113 (4.0%)	84 (4.0%)	29 (3.8%)	
Lung- 2nd line immunotherapy	994 (35.0%)	732 (35.2%)	262 (34.5%)	
Bladder	105 (3.7%)	77 (3.7%)	28 (3.7%)	
Pancreas	753 (26.5%)	551 (26.5%)	202 (26.6%)	
Colon	181 (6.4%)	134 (6.4%)	47 (6.2%)	
Kidney	155 (5.5%)	115 (5.5%)	40 (5.3%)	
Breast- first line	376 (13.2%)	273 (13.1%)	103 (13.6%)	
Breast- second line	91 (3.2%)	65 (3.1%)	26 (3.4%)	
Melanoma	50-60 (1.0-3.0%)	30-40 (1.0-2.0%)	10-20 (1.0-3.0%)	
Time period				0.04
Q1/2 2015	97 (3.4%)	70 (3.4%)	27 (3.6%)	
Q3/4 2015	299 (10.5%)	215 (10.3%)	84 (11.1%)	
Q1/2 2016	882 (31.1%)	645 (31.0%)	237 (31.2%)	
Q3/4 2016	797 (28.1%)	589 (28.3%)	208 (27.4%)	
Q1/2 2017	496 (17.5%)	365 (17.6%)	131 (17.2%)	
Q3/4 2017	220 (7.7%)	162 (7.8%)	58 (7.6%)	
Q1/2 2018	48 (1.7%)	33 (1.6%)	15 (2.0%)	
Urbanicity				0.19
Metro	2,423 (85.3%)	1,739 (83.6%)	684 (90.0%)	
Non-metro	416 (14.7%)	340 (16.4%)	76 (10.0%)	
Census tract poverty rate				0.24
0% - <5%	689 (24.3%)	457 (22.0%)	232 (30.5%)	
5% - <10%	672 (23.7%)	478 (23.0%)	194 (25.5%)	
10% - <155%	801 (28.2%)	620 (29.8%)	181 (23.8%)	
15% - <20%	473 (16.7%)	360 (17.3%)	113 (14.9%)	
Unknown	204 (7.2%)	164 (7.9%)	40 (5.3%)	
Treating oncologist				0.04
Low volume	32 (1.1%)	20-30 (1.0-3.0%)	<11 (0-2%)	

© 2022 Manz CR et al. *JAMA Network Open.*

Specialist	1,067 (37.6%)	760-770 (36.0-37.0%)	290-300 (38.5-39.5%)	
Generalist	1,740 (61.3%)	1,286 (61.9%)	454 (59.7%)	

** Ranges provided for some values to comply with CMS's cell suppression policy.*

© 2022 Manz CR et al. *JAMA Network Open.*

eTable 4. Receipt of Novel Therapies by Oncology Care Model Status, Before and After Intervention, Black vs White Patients

	**Pre-OCM (% receiving novel therapy)**	**Post-OCM (% receiving novel therapy)**	**Adjusted Difference-in-Differences (percentage points)***	**Adjusted Triple differences (percentage points)**
White patients				
OCM	40.8	49.9	9.1	
Non-OCM	33.7	41.1	7.4	
			1.8	
Black patients				
OCM	27.8	54.1	26.3	
Non-OCM	25.8	29.0	3.2	
			23.0	
				21.2
** Differences may not add up due to rounding.*				

© 2022 Manz CR et al. *JAMA Network Open.*

eAppendix 1. Parallel trends regression output

```
Mixed-effects logistic regression               Number of obs     =      1,273
Group variable:        _MatchID                 Number of groups  =        348

                                                Obs per group:
                                                              min =          2
                                                              avg =        3.7
                                                              max =          4

Integration method: mvaghermite                 Integration pts.  =          7

                                                Wald chi2(30)     =     176.99
Log likelihood = -645.25964                     Prob > chi2       =     0.0000
------------------------------------------------------------------------------------------------
                      outcome | Odds Ratio   Std. Err.      z    P>|z|     [95% Conf. Interval]
------------------------------+-----------------------------------------------------------------
                      Quarter |   1.559896   .1350359     5.14   0.000     1.316465     1.84834
                              |
                       altocm |
                         OCM  |   9.421751   11.23917     1.88   0.060     .9093619    97.61723
                              |
               altocm#c.Quarter |
                         OCM  |   .8132178   .1084143    -1.55   0.121     .6262227    1.056051
                              |
                       agegrp |
                       65-69  |   .9557352   .2769244    -0.16   0.876      .541629    1.686449
                       70-74  |   1.003951    .285512     0.01   0.989     .5749644     1.75301
                       75-79  |   1.549284     .44962     1.51   0.131     .8772083    2.736274
                         80+  |   .8169643   .2535128    -0.65   0.515     .4446989     1.50086
                              |
                        1.SEX |    1.12848   .1863739     0.73   0.464     .8164201    1.559818
                              |
                         race |
                       Black  |   .5837749   .1679906    -1.87   0.061     .3321227    1.026106
Asian, Pacific Islander, Ot..  |   1.046783   .3078007     0.16   0.876     .5882547     1.86272
                              |
                   1.hispanic |   1.455724   .4681644     1.17   0.243     .7750511    2.734182
                              |
                     marriage |
                     Married  |   1.094645   .1792573     0.55   0.581      .794112    1.508914
                     Unknown  |   .8041205   .2458542    -0.71   0.476      .441644    1.464098
                              |
                   urbanrural |
                   Non-metro  |   .8688095   .1873492    -0.65   0.514     .5693408    1.325796
                              |
                      poverty |
                  5% - <10%   |   .8128745   .1697673    -0.99   0.321     .5398234    1.224039
                10% - <155%   |   .7491853   .1550072    -1.40   0.163     .4994302    1.123838
                 15% - <20%   |   .7926447   .1912014    -0.96   0.335     .4940286     1.27176
                     Unknown  |   .6806882    .230282    -1.14   0.256     .3507359    1.321041
                              |
                       cohort |
                  Lung- EGFR  |    .331775   .3200302    -1.14   0.253     .0500927    2.197419
 Lung- 2nd line immunotherapy  |   .2781601   .2523249    -1.41   0.158     .0470058     1.64603
                    Pancreas  |   .0340452   .0317847    -3.62   0.000     .0054623    .2121976
                       Colon  |    .016367    .016739    -4.02   0.000     .0022051    .1214826
                      Kidney  |   1.777283   2.054928     0.50   0.619     .1843237    17.13688
          Breast- first line  |   .1710546   .1573354    -1.92   0.055      .028197    1.037688
         Breast- second line  |   .1831655   .1985586    -1.57   0.117     .0218831    1.533132
                    Melanoma  |   1.576144   1.625636     0.44   0.659     .2087688    11.89942
                              |
                     Charlson |
                           1  |   .8818282   .1542877    -0.72   0.472     .6258278    1.242548
                          >2  |   .7985992   .1409367    -1.27   0.203     .5650787    1.128623
                              |
                providerclass |
                  Specialist  |   .4453698   .3185495    -1.13   0.258     .1096229    1.809423
                 Generalist  |   .4274224   .3036447    -1.20   0.232     .1062077    1.720119
```

© 2022 Manz CR et al. *JAMA Network Open.*

```
                                 |
                           _cons |   .1266129   .1777107    -1.47   0.141     .0080864    1.982455
---------------------------------+----------------------------------------------------------------
_MatchID                         |
                      var(_cons)|   .1919462   .1679595                      .0345421     1.06662
--------------------------------------------------------------------------------------------------
Note: Estimates are transformed only in the first equation.
Note: _cons estimates baseline odds (conditional on zero random effects).
LR test vs. logistic model: chibar2(01) = 1.63        Prob >= chibar2 = 0.1009
```

© 2022 Manz CR et al. *JAMA Network Open.*

eAppendix 2. Difference-in-differences regression output

```
Mixed-effects logistic regression               Number of obs     =      2,833
Group variable:        _MatchID                 Number of groups  =        760

                                                Obs per group:
                                                              min =          2
                                                              avg =        3.7
                                                              max =          4

Integration method: mvaghermite                 Integration pts.  =          7

                                                Wald chi2(31)     =     383.07
Log likelihood = -1557.3771                     Prob > chi2       =     0.0000
------------------------------------------------------------------------------------------------
                    outcome | Odds Ratio   Std. Err.      z    P>|z|     [95% Conf. Interval]
----------------------------+-------------------------------------------------------------------
                    postOCM |
                      Post  |   1.479894   .1785226     3.25   0.001     1.168284    1.874618
                            |
                     altocm |
                       OCM  |   1.466395   .2209095     2.54   0.011     1.091489    1.970075
                            |
             postOCM#altocm |
                  Post#OCM  |   1.185962   .2394865     0.84   0.398     .7983328    1.761804
                            |
                     agegrp |
                     65-69  |   .9440269   .1739832    -0.31   0.755     .6578226    1.354753
                     70-74  |   1.083553   .1984944     0.44   0.661     .7566933    1.551602
                     75-79  |   1.389753    .263565     1.74   0.083     .9583145    2.015426
                       80+  |   1.154709   .2277148     0.73   0.466     .7845325     1.69955
                            |
                      1.SEX |   1.026918   .1066806     0.26   0.798     .8377399    1.258816
                            |
                       race |
                     Black  |   .6119065   .1153714    -2.61   0.009     .4228593    .8854707
Asian, Pacific Islander, Ot..  |   1.041884   .1976893     0.22   0.829     .7183079    1.511222
                            |
                 1.hispanic |   .9312589   .1868228    -0.36   0.723     .6285042    1.379853
                            |
                   marriage |
                   Married  |   1.002412   .1061081     0.02   0.982     .8145998    1.233526
                   Unknown  |   .6366298   .1118337    -2.57   0.010      .451191    .8982837
                            |
                 urbanrural |
                 Non-metro  |   .8899028   .1250653    -0.83   0.407     .6756414    1.172111
                            |
                    poverty |
                 5% - <10%  |   .8748658   .1159094    -1.01   0.313     .6747879    1.134268
               10% - <155%  |   .8533478    .111095    -1.22   0.223      .661166    1.101391
                15% - <20%  |   .8359725   .1313468    -1.14   0.254     .6144016    1.137448
                   Unknown  |    .722856   .1508223    -1.56   0.120     .4802313    1.088061
                            |
                     cohort |
                Lung- EGFR  |   .8275649   .5850669    -0.27   0.789     .2070259    3.308107
 Lung- 2nd line immunotherapy  |   .7312314   .4925701    -0.46   0.642     .1952859    2.738034
                   Bladder  |   3.460971   2.545082     1.69   0.091     .8189416    14.62659
                  Pancreas  |     .16025   .1089533    -2.69   0.007     .0422737    .6074718
                     Colon  |   .0318576   .0244428    -4.49   0.000     .0070816    .1433172
                    Kidney  |   2.479545   1.756236     1.28   0.200     .6186923    9.937319
        Breast- first line  |   .2293813   .1569131    -2.15   0.031     .0600172    .8766787
       Breast- second line  |   1.037199   .7445973     0.05   0.959     .2539749    4.235778
                  Melanoma  |   5.140568   4.065018     2.07   0.038     1.091201    24.21685
                            |
                   Charlson |
                         1  |   .7955131    .091266    -1.99   0.046     .6353199    .9960983
                        >2  |   .8164413   .0922566    -1.79   0.073     .6542452    1.018848
                            |
```

© 2022 Manz CR et al. *JAMA Network Open.*

```
                providerclass |
                  Specialist  |   .7379821   .3399949    -0.66   0.510     .2991505    1.820547
                  Generalist  |   .7268647   .3331315    -0.70   0.486     .2960308    1.784721
                              |
                        _cons |   1.676043   1.405667     0.62   0.538       .32389    8.673068
------------------------------+----------------------------------------------------------------
_MatchID                      |
                   var(_cons)|   .2730109   .1097865                      .1241321    .6004485
-----------------------------------------------------------------------------------------------
Note: Estimates are transformed only in the first equation.
Note: _cons estimates baseline odds (conditional on zero random effects).
LR test vs. logistic model: chibar2(01) = 8.46        Prob >= chibar2 = 0.0018


Predictive margins                              Number of obs     =      2,833
Model VCE    : OIM

Expression   : Marginal predicted mean, predict()

--------------------------------------------------------------------------------
              |            Delta-method
              |     Margin   Std. Err.      z    P>|z|     [95% Conf. Interval]
--------------+-----------------------------------------------------------------
postOCM#altocm |
  Pre#Non-OCM  |   .3315077   .0151807    21.84   0.000     .3017541    .3612613
      Pre#OCM  |   .3989276   .0238513    16.73   0.000     .3521799    .4456752
 Post#Non-OCM  |   .4005897   .0139109    28.80   0.000     .3733247    .4278547
     Post#OCM  |   .5031169   .0224456    22.41   0.000     .4591244    .5471094
--------------------------------------------------------------------------------


Contrasts of average marginal effects
Model VCE    : OIM

Expression   : Marginal predicted mean, predict()
dy/dx w.r.t. : 1.postOCM

------------------------------------------------
             |         df        chi2     P>chi2
-------------+----------------------------------
0b.postOCM   |
      altocm |  (omitted)
-------------+----------------------------------
1.postOCM    |
      altocm |          1        0.92     0.3378
------------------------------------------------

-----------------------------------------------------------------
                  |   Contrast Delta-method
                  |      dy/dx   Std. Err.     [95% Conf. Interval]
------------------+----------------------------------------------
0.postOCM         |  (base outcome)
------------------+----------------------------------------------
1.postOCM         |
           altocm |
(OCM vs Non-OCM)  |   .0351073   .0366303     -.0366868    .1069015
-----------------------------------------------------------------
Note: dy/dx for factor levels is the discrete change from the base
      level.
```

© 2022 Manz CR et al. *JAMA Network Open.*

eAppendix 3. Difference-in-differences regression output for second-line immunotherapy in lung cancer cohort

```
Mixed-effects logistic regression               Number of obs     =        994
Group variable:        _MatchID                 Number of groups  =        262

                                                Obs per group:
                                                              min =          2
                                                              avg =        3.8
                                                              max =          4

Integration method: mvaghermite                 Integration pts.  =          7

                                                Wald chi2(22)     =      94.39
Log likelihood = -624.83618                     Prob > chi2       =     0.0000
```

outcome	Odds Ratio	Std. Err.	z	P>|z|	[95% Conf.	Interval]
postOCM						
Post	1.308788	.2366481	1.49	0.137	.9182473	1.865431
altocm						
OCM	1.494058	.3226243	1.86	0.063	.9784988	2.281258
postOCM#altocm						
Post#OCM	2.462592	.8215845	2.70	0.007	1.280589	4.735604
agegrp						
65-69	.9145318	.2590876	-0.32	0.752	.5248711	1.593474
70-74	1.43883	.4093878	1.28	0.201	.8237942	2.513046
75-79	2.696624	.801521	3.34	0.001	1.505972	4.828632
80+	2.562848	.8253005	2.92	0.003	1.363374	4.817601
1.SEX	1.012876	.1548094	0.08	0.933	.7506847	1.366643
race						
Black	.6566339	.1881604	-1.47	0.142	.374463	1.15143
Asian, Pacific Islander, Ot..	1.123937	.3439697	0.38	0.703	.6169362	2.047593
1.hispanic	.644373	.2452557	-1.15	0.248	.3056049	1.358671
marriage						
Married	.9602361	.1571842	-0.25	0.804	.696694	1.32347
Unknown	.2740326	.1258382	-2.82	0.005	.1114096	.6740336
urbanrural						
Non-metro	.5351051	.1184699	-2.82	0.005	.3467264	.8258312
poverty						
5% - <10%	.7557594	.1649014	-1.28	0.199	.4927849	1.15907
10% - <155%	.9332617	.1966738	-0.33	0.743	.61748	1.410536
15% - <20%	.8617498	.2136736	-0.60	0.548	.5300575	1.401004
Unknown	.743602	.2380689	-0.93	0.355	.3970296	1.392702
Charlson						
1	.6945171	.1284472	-1.97	0.049	.4833456	.9979485
>2	.6780703	.1227965	-2.15	0.032	.4754717	.9669961
providerclass						
Specialist	.3585782	.2997138	-1.23	0.220	.0696813	1.845235
Generalist	.4731749	.3921597	-0.90	0.367	.0932309	2.401506
_cons	1.875952	1.679707	0.70	0.482	.3243883	10.84871
_MatchID						
var(_cons)	.3807026	.186039			.1460921	.9920759

Note: Estimates are transformed only in the first equation.
Note: _cons estimates baseline odds (conditional on zero random effects).
LR test vs. logistic model: chibar2(01) = 6.44   Prob >= chibar2 = 0.0056

© 2022 Manz CR et al. *JAMA Network Open.*

```
. margins postOCM#altocm

Predictive margins                              Number of obs     =        994
Model VCE    : OIM

Expression   : Marginal predicted mean, predict()

--------------------------------------------------------------------------------
               |            Delta-method
               |     Margin   Std. Err.      z    P>|z|     [95% Conf. Interval]
---------------+----------------------------------------------------------------
postOCM#altocm |
  Pre#Non-OCM  |   .4184403   .0259974    16.10   0.000     .3674864    .4693943
      Pre#OCM  |   .5034004   .0407281    12.36   0.000     .4235748    .5832259
 Post#Non-OCM  |   .4751974   .0276395    17.19   0.000     .4210251    .5293698
     Post#OCM  |   .7346387   .0392178    18.73   0.000     .6577734    .8115041
--------------------------------------------------------------------------------

. margins r.altocm, dydx(postOCM)

Contrasts of average marginal effects
Model VCE    : OIM

Expression   : Marginal predicted mean, predict()
dy/dx w.r.t. : 1.postOCM

------------------------------------------------
             |         df        chi2     P>chi2
-------------+----------------------------------
0b.postOCM   |
      altocm |  (omitted)
-------------+----------------------------------
1.postOCM    |
      altocm |          1        7.33     0.0068
------------------------------------------------

-----------------------------------------------------------------
                  |   Contrast Delta-method
                  |      dy/dx   Std. Err.     [95% Conf. Interval]
------------------+----------------------------------------------
0.postOCM         |  (base outcome)
------------------+----------------------------------------------
1.postOCM         |
           altocm |
(OCM vs Non-OCM)  |   .1744812   .0644641      .0481338    .3008286
-----------------------------------------------------------------
Note: dy/dx for factor levels is the discrete change from the base
      level.
```

© 2022 Manz CR et al. *JAMA Network Open.*

eAppendix 4. Difference-in-difference-in-differences regression output for race

```
Mixed-effects logistic regression               Number of obs     =      2,833
Group variable:        _MatchID                 Number of groups  =        760

                                                Obs per group:
                                                              min =          2
                                                              avg =        3.7
                                                              max =          4

Integration method: mvaghermite                 Integration pts.  =          7

                                                Wald chi2(37)     =     384.51
Log likelihood = -1555.6654                     Prob > chi2       =     0.0000
------------------------------------------------------------------------------------------
                       outcome | Odds Ratio   Std. Err.      z    P>|z|     [95% Conf. Interval]
-------------------------------+----------------------------------------------------------
                       postOCM |
                         Post  |   1.513759   .1951955     3.22   0.001     1.175699    1.949023
                               |
                        altocm |
                          OCM  |   1.488762   .2417192     2.45   0.014     1.082984    2.046579
                               |
                 postOCM#altocm |
                     Post#OCM  |   1.078996   .2337956     0.35   0.726     .7056389    1.649899
                               |
                        agegrp |
                        65-69  |   .9432606   .1740811    -0.32   0.752     .6569622    1.354325
                        70-74  |   1.082179   .1986774     0.43   0.667     .7551388    1.550855
                        75-79  |   1.386539      .2634     1.72   0.085     .9554985     2.01203
                          80+  |   1.159404   .2291934     0.75   0.454     .7869867    1.708056
                               |
                         1.SEX |   1.033377   .1075073     0.32   0.752     .8427603    1.267108
                               |
                          race |
                        Black  |    .613715   .1980646    -1.51   0.130     .3260303    1.155249
Asian, Pacific Islander, Ot..  |   1.082346   .3511448     0.24   0.807      .573075    2.044188
                               |
                   race#altocm |
                    Black#OCM  |   .7679486   .4675071    -0.43   0.664     .2328857    2.532337
Asian, Pacific Islander, Ot.. #|
                          OCM  |    1.07821   .7153948     0.11   0.910     .2937173    3.958016
                               |
                  race#postOCM |
                   Black#Post  |   .8101041   .3478427    -0.49   0.624     .3491819    1.879446
Asian, Pacific Islander, Ot.. #|
                         Post  |   .8803851   .3666783    -0.31   0.760     .3891789    1.991572
                               |
           race#altocm#postOCM |
               Black#OCM#Post  |   3.270622   2.665859     1.45   0.146     .6619458    16.15989
Asian, Pacific Islander, Ot.. #|
                          OCM #|
                         Post  |   1.171238   1.093088     0.17   0.866     .1880352    7.295434
                               |
                    1.hispanic |   .9285109   .1864045    -0.37   0.712     .6264738    1.376167
                               |
                      marriage |
                      Married  |   1.004519   .1064429     0.04   0.966     .8161343    1.236389
                      Unknown  |   .6386162   .1122437    -2.55   0.011      .452514    .9012553
                               |
                    urbanrural |
                    Non-metro  |   .8839386    .124447    -0.88   0.381      .670786    1.164824
                               |
                       poverty |
                   5% - <10%   |    .873471   .1158545    -1.02   0.308     .6735157     1.13279
                 10% - <155%   |   .8559778   .1116064    -1.19   0.233     .6629472    1.105213
                  15% - <20%   |   .8362434   .1315826    -1.14   0.256     .6143225    1.138332
                      Unknown  |   .7268139   .1516156    -1.53   0.126     .4829031    1.093922
```

© 2022 Manz CR et al. *JAMA Network Open.*

```
                                |
                         cohort |
                     Lung- EGFR  |   .8273083   .5844653    -0.27   0.788     .2071678    3.30379
 Lung- 2nd line immunotherapy  |   .7256062   .4882887    -0.48   0.634     .1940413   2.713362
                       Bladder  |   3.495794   2.568879     1.70   0.089     .8280218   14.75876
                      Pancreas  |   .1595215   .1083564    -2.70   0.007      .042134   .6039562
                         Colon  |   .0317294   .0243308    -4.50   0.000      .007059     .14262
                        Kidney  |   2.468409   1.747143     1.28   0.202     .6165034   9.883224
             Breast- first line  |   .2268545     .15505    -2.17   0.030     .0594251   .8660135
            Breast- second line  |   1.009606    .724393     0.01   0.989     .2474084   4.119928
                      Melanoma  |   5.142736   4.064149     2.07   0.038     1.092736   24.20322
                                |
                       Charlson |
                             1  |   .7971792   .0915246    -1.97   0.048     .6365448   .9983502
                            >2  |   .8205413   .0929697    -1.75   0.081     .6571384   1.024576
                                |
                  providerclass |
                    Specialist  |   .7140769   .3295444    -0.73   0.466     .2890133   1.764298
                    Generalist  |   .7042277   .3232721    -0.76   0.445     .2864003   1.731621
                                |
                          _cons |   1.726761   1.448357     0.65   0.515     .3336331   8.937073
--------------------------------+----------------------------------------------------------------
_MatchID                        |
                     var(_cons)|   .2724887   .1101477                        .1233868    .601767
-------------------------------------------------------------------------------------------------
Note: Estimates are transformed only in the first equation.
Note: _cons estimates baseline odds (conditional on zero random effects).
LR test vs. logistic model: chibar2(01) = 8.37        Prob >= chibar2 = 0.0019


Contrasts of average marginal effects
Model VCE    : OIM

Expression   : Marginal predicted mean, predict()
dy/dx w.r.t. : 1.postOCM

1._at        : race            =           1

2._at        : race            =           2

-------------------------------------------------------
                    |         df        chi2     P>chi2
--------------------+----------------------------------
0b.postOCM          |
         altocm@_at |
(OCM vs Non-OCM) 1  |  (omitted)
(OCM vs Non-OCM) 2  |  (omitted)
             Joint  |  (omitted)
--------------------+----------------------------------
1.postOCM           |
         altocm@_at |
(OCM vs Non-OCM) 1  |          1        0.20     0.6584
(OCM vs Non-OCM) 2  |          1        3.14     0.0766
             Joint  |          2        3.35     0.1873
-------------------------------------------------------

-------------------------------------------------------------------------------------
                    |   Contrast
                    |      dy/dx  Legend
--------------------+----------------------------------------------------------------
0.postOCM           |  (base outcome)
--------------------+----------------------------------------------------------------
1.postOCM           |
         altocm@_at |
(OCM vs Non-OCM) 1  |   .0175164  _b[1.postOCM:r2vs1.altocm@1bn._at]
(OCM vs Non-OCM) 2  |   .2304112  _b[1.postOCM:r2vs1.altocm@2._at]
-------------------------------------------------------------------------------------
Note: dy/dx for factor levels is the discrete change from the base level.
```

© 2022 Manz CR et al. *JAMA Network Open.*

```
. nlcom (_b[1.postOCM:r2vs1.altocm@2._at] - _b[1.postOCM:r2vs1.altocm@1bn._at])

       _nl_1:  _b[1.postOCM:r2vs1.altocm@2._at] - _b[1.postOCM:r2vs1.altocm@1bn._at]

------------------------------------------------------------------------------
             |      Coef.   Std. Err.      z    P>|z|     [95% Conf. Interval]
-------------+----------------------------------------------------------------
       _nl_1 |   .2128948   .1364749     1.56   0.119    -.0545911    .4803807
------------------------------------------------------------------------------
```

© 2022 Manz CR et al. *JAMA Network Open.*

eAppendix 5. Regression output for sensitivity analysis of primary difference-in-differences model using alternative specification of Oncology Care Model practices

```
Mixed-effects logistic regression               Number of obs     =      1,730
Group variable:        _MatchID                 Number of groups  =        440

                                                Obs per group:
                                                              min =          2
                                                              avg =        3.9
                                                              max =          4

Integration method: mvaghermite                 Integration pts.  =          7

                                                Wald chi2(31)     =     242.45
Log likelihood =  -969.4363                     Prob > chi2       =     0.0000
```

outcome	Odds Ratio	Std. Err.	z	P>|z|	[95% Conf.	Interval]
postOCM						
Post	1.391171	.215052	2.14	0.033	1.027541	1.883485
altocm						
OCM	1.760338	.3260981	3.05	0.002	1.224371	2.530923
postOCM#altocm						
Post#OCM	.9416811	.2329019	-0.24	0.808	.5799357	1.529072
agegrp						
65-69	1.000938	.2397592	0.00	0.997	.6259145	1.600661
70-74	1.173409	.2779729	0.68	0.500	.7375736	1.866782
75-79	1.469289	.3599746	1.57	0.116	.9089996	2.374931
80+	1.162009	.2943392	0.59	0.553	.7072916	1.909064
1.SEX	1.093049	.1425179	0.68	0.495	.8465553	1.411316
race						
Black	.6184799	.1461739	-2.03	0.042	.389179	.9828831
Asian, Pacific Islander, Ot..	.9574592	.2193617	-0.19	0.850	.611087	1.50016
1.hispanic	.8365471	.2150725	-0.69	0.488	.5054175	1.38462
marriage						
Married	1.051766	.1405491	0.38	0.706	.8094159	1.366679
Unknown	.8511737	.1866229	-0.73	0.462	.5538462	1.308119
urbanrural						
Non-metro	.9890494	.1832515	-0.06	0.953	.687871	1.422096
poverty						
5% - <10%	.991822	.1621312	-0.05	0.960	.7199286	1.366401
10% - <155%	.7961236	.1293328	-1.40	0.160	.5790288	1.094614
15% - <20%	.835205	.1669417	-0.90	0.368	.5644871	1.235754
Unknown	.8026243	.2130316	-0.83	0.407	.4770757	1.350322
cohort						
Lung- EGFR	2.787101	2.055965	1.39	0.165	.6565161	11.83205
Lung- 2nd line immunotherapy	1.846352	1.301173	0.87	0.384	.4639277	7.34816
Bladder	8.183314	6.466757	2.66	0.008	1.738915	38.51059
Pancreas	.3999201	.2844241	-1.29	0.198	.0992195	1.611943
Colon	.1040871	.0854264	-2.76	0.006	.0208352	.5199905
Kidney	5.326752	3.962288	2.25	0.025	1.239649	22.88896
Breast- first line	.5108939	.3678605	-0.93	0.351	.1245768	2.095193
Breast- second line	2.147256	1.628335	1.01	0.314	.4857239	9.492443
Melanoma	5.912005	4.728024	2.22	0.026	1.233108	28.34448
Charlson						
1	.8875772	.1265095	-0.84	0.403	.6712455	1.173629
>2	.9214635	.1316268	-0.57	0.567	.6964469	1.219181

© 2022 Manz CR et al. *JAMA Network Open.*

```
                            |
              providerclass |
                Specialist  |   .7021813   .4836265    -0.51   0.608     .1820477    2.708403
                Generalist  |    .686668    .472526    -0.55   0.585     .1782371    2.645425
                            |
                      _cons |   .5962869   .6045658    -0.51   0.610     .0817402    4.349857
----------------------------+----------------------------------------------------------------
_MatchID                    |
                 var(_cons)|   .1914421   .1259693                      .0527163    .6952323
---------------------------------------------------------------------------------------------
Note: Estimates are transformed only in the first equation.
Note: _cons estimates baseline odds (conditional on zero random effects).
LR test vs. logistic model: chibar2(01) = 2.90        Prob >= chibar2 = 0.0442
```

```
Predictive margins                              Number of obs     =      1,730
Model VCE    : OIM

Expression   : Marginal predicted mean, predict()

--------------------------------------------------------------------------------
              |            Delta-method
              |     Margin   Std. Err.      z    P>|z|     [95% Conf. Interval]
--------------+-----------------------------------------------------------------
postOCM#altocm |
  Pre#Non-OCM  |   .3398412   .0203402    16.71   0.000     .2999751    .3797074
      Pre#OCM  |   .4437285   .0291768    15.21   0.000     .3865431    .5009139
 Post#Non-OCM  |   .3995796   .0178962    22.33   0.000     .3645037    .4346554
     Post#OCM  |   .4951722   .0272047    18.20   0.000      .441852    .5484925
--------------------------------------------------------------------------------
```

```
Contrasts of average marginal effects
Model VCE    : OIM

Expression   : Marginal predicted mean, predict()
dy/dx w.r.t. : 1.postOCM

------------------------------------------------
             |         df        chi2     P>chi2
-------------+----------------------------------
0b.postOCM   |
      altocm |  (omitted)
-------------+----------------------------------
1.postOCM    |
      altocm |          1        0.03     0.8575
------------------------------------------------

-------------------------------------------------------------------
                  |   Contrast Delta-method
                  |      dy/dx   Std. Err.     [95% Conf. Interval]
------------------+------------------------------------------------
0.postOCM         |  (base outcome)
------------------+------------------------------------------------
1.postOCM         |
           altocm |
(OCM vs Non-OCM)  |  -.0082946   .0462093     -.0988632    .0822739
-------------------------------------------------------------------
Note: dy/dx for factor levels is the discrete change from the base
      level.
```

© 2022 Manz CR et al. *JAMA Network Open.*
